# CA 19-9 but Not IGF-1/IGFBP-2 Is a Useful Biomarker for Pancreatic Ductal Adenocarcinoma (PDAC) and Chronic Pancreatitis (CP) Differentiation

**DOI:** 10.3390/jcm12124050

**Published:** 2023-06-14

**Authors:** Barbara Wlodarczyk, Lukasz Durko, Przemyslaw Wlodarczyk, Renata Talar-Wojnarowska, Ewa Malecka-Wojciesko

**Affiliations:** 1 Department of Digestive Tract Diseases, Medical University of Lodz, 90-419 Lodz, Poland; 2 Faculty of Economics and Sociology, University of Lodz, 90-419 Lodz, Poland

**Keywords:** pancreatic cancer, chronic pancreatitis, insulin-like growth factor 1, insulin-like growth factor-binding protein 2

## Abstract

Introduction: There are still no effective diagnostic and prognostic biomarkers in pancreatic ductal adenocarcinoma (PDAC). The differentiation between PDAC and chronic pancreatitis (CP) is often challenging. The inflammatory mass in the course of CP causes diagnostic difficulties in differentiating them from neoplastic lesions and, thus, delays the initiation of radical treatment. Insulin-like growth factor 1 (IGF-1) and insulin-like growth factor-binding protein 2 (IGFBP-2) form a network involved in PDAC development. The role of IGFs in promoting pancreatic cancer cell proliferation, survival, and migration is well established, and their ability to stimulate tumor growth and metastasis is well documented. The aim of the study was to evaluate the usability of IGF-1, IGFBP-2, and IGF-1/IGFBP-2 ratio in PDAC and CP differentiation. Material and methods: The study included 137 patients: 89 patients with PDAC and 48 patients with CP. All subjects were tested for the levels of IGF-1 and IGFBP-2 using the ELISA method (Corgenix UK Ltd. R&D Systems), along with the level of CA 19-9 in serum. Additionally, the IGF-1/IGFBP-2 ratio was calculated. Further analyses used logit and probit models with varying determinants in order to discern between PDAC and CP patients. The models served as a basis for AUROC calculation. Results: The mean IGF-1 serum level was equal to 52.12 ± 33.13 ng/mL in PDAC vs. 74.23 ± 48.98 ng/mL in CP (*p* = 0.0053). The mean level of IGFBP-2 was equal to 305.95 ± 194.58 ng/mL in PDAC vs. 485.43 ± 299 ng/mL in CP (*p* = 0.0002). The mean CA 19-9 serum concentration was 434.95 ± 419.98 U/mL in PDAC vs. 78.07 ± 182.36 U/mL in CP (*p* = 0.0000). The mean IGF-1/IGFBP-2 ratio was 0.213 ± 0.14 in PDAC vs. 0.277 ± 0.33 in CP (*p* = 0.1914). The diagnostic usefulness of indicators for the purpose of PDAC and CP differentiation was assessed by means of AUROC comparison. The AUROCs of IGF-1, IGFBP-2, and IGF-1/IGFBP-2 ratio ranged below 0.7, being lower than the AUROC of CA 19-9 (0.7953; 0.719 within 95% CI). Together, the CA 19-9 and IGFBP-2 AUROCs also ranged below 0.8. When age was included, the AUROC increased to 0.8632, and its 95% confidence interval held above the 0.8 limit. The sensitivity of the used markers was not correlated to the stage of pancreatic PDAC. Conclusions: The presented results indicate that CA 19-9 is a marker demonstrating high potential for PDAC and CP differentiation. The inclusion of additional variables into the model, such as the serum level of IGF-1 or IGFBP-2, slightly increased the sensitivity in differentiating CP from PDAC. The IGF-1/IGFBP-2 ratio turned out to be a good marker of pancreatic diseases, but insufficient for the purpose of CP and PDAC differentiation.

## 1. Introduction

Pancreatic ductal adenocarcinoma (PDAC) has one of the worst 5 year prognoses among cancer types, estimated at about 5% [1]. It is associated with the late onset of nonspecific symptoms, such as unintentional weight loss. Its poor survival rate is mainly related to its aggressive behavior. When PDAC is detected, resection is possible only in 10–20% of cases, which is closely related to tumor stage [2]. The dense fibroblastic stroma bordering the tumor is characteristic for PDAC. The presence of inflammatory and immune cells, fibroblasts, and pancreatic stellate cells (PaSCs) is crucial in the development of PDAC, in addition to an increased level of extracellular matrix (ECM) components [3,4]. The progression of pancreatic intraepithelial neoplasia (PanIN) lesions to PDAC is associated with changes in the stroma and desmoplastic responses [5,6].

Hyperinsulinemia and insulin resistance are representative endocrine disorders in PDAC. In this metabolic state, insulin increases the accessibility of factors, i.e., the insulin-like growth factor axis, regulating metabolism and growth [7]. The IGF axis consists of insulin, insulin-like growth factors 1 and 2 (IGF-1 and 2), receptors (IGF-R), IGF-binding proteins 1–7 (IGFBPs), and IGFBP proteases. Among the IGFs, the IGF-1 gene encodes a protein consisting of 70 amino-acid residues. It is located on chromosome 12q23-23 [8]. IGF-1 is an anabolic insulin-like protein which is stimulated by growth hormone (GH) and is the major mediator of GH effects [9,10]. β-cells in the pancreas produce insulin, while IGF-1 and IGF-2 are released by hepatocytes in response to GH stimulation [11]. The functions of IGF-1 mostly involve mediating insulin sensitivity, glucose uptake, and triglyceride level [12]. IGF-1 is in control of the hormonal balance between insulin and GH [13]. The deterioration of IGF-1 synthesis aggravates the state of insulin resistance [12].

Insulin resistance, hyperinsulinemia, hyperglycemia, and elevated levels of IGF-1 activate PaSCs that express IGF-R [14]. Correlated with mitogenic and antiapoptotic effects, a higher level of IGF-1 is connected with increased risk for PDAC [11,15,16,17,18,19]. PaSCs derived from PDAC and their migratory ability are also stimulated by IGF-1 changes [20,21]. The PDAC stroma is composed of “acellular” ingredients, i.e., extracellular matrix proteins such as collagen, fibronectin, or laminin. The “cellular” part consists of the factors of the PDAC microenvironment such as immune cells, endothelial cells, PaSCs, fibroblasts, or neural cells [22]. To sum up, the insulin/IGF-1R pathway is a link between cancer cells and the stroma. Moreover, stromal cells such as cancer-associated fibroblasts (CAFs) and tumor-associated macrophages are major sources of tumor-derived IGF-1 in PDAC [23,24]. Furthermore, stromal cells produce proteases to cleave IGFBPs, enhancing the bioavailability of IGF-1 [25]. Tumor- and stromal-derived IGF-1 stimulates signaling, which increases cell proliferation and survival while reducing apoptosis [26,27]. The insulin/IGF interplay among PDAC cells, the pancreas, and the stroma may be a key to understanding the development of pancreatic diabetes [28]. Research evidence has disclosed that the IGF and IGFBP axis facilitates PDAC development, metastasis, and drug resistance, and it may promote tumor progression into an advanced stage [26,29,30]. Due to the participation of the IGF axis in the progression of PDAC, we previously demonstrated the usefulness of IGF proteins as biomarkers of early pancreatic cancer [31,32,33].

Mutgan et al., on the basis of other studies, proposed five pieces of evidence indicating the role of stromal IGF signaling in PDAC development [28]. Firstly, pancreatic stromal cells or fibroblasts secrete IGF-1 and increase the migration position of PDAC cells [21,34]. Secondly, proteases such as metalloproteinases-3, -7, or -9 (MMP-3, MMP-7, or MMP-9) secreted by stromal cells can cleave IGFBPs and raise the IGF activity [25,35]. Then, pancreatic tumor growth can also be controlled by chemokines, stromal cells, and immune cells such as macrophages [27]. Next, hyperglycemia activates stromal cells, causing fibrosis of the pancreatic islets and increasing endocrine disorders [36]. Lastly, endocrine or exocrine progenitors (e.g., Neruogenin 3, Pax6, MafA, and Pdx1) causing trans-differentiation of cells are stimulated by progressive fibrosis in pancreatic tissue [37].

Binding proteins and IGF ligands bind to their receptors with high affinity. IGF-1 and IGFBP-2 may have increased bioavailability due to the presence of proteases that cleave IGFBPs from their ligands [38]. The main role of IGFBPs is to protect IGFs from destruction by creating the IGF–IGFBP ligand. IGFBPs consist of 200–300 amino acids and various domains: disulfide-constrained cysteine-rich amino-terminal and evolutionarily conserved cysteine-rich carboxy-terminal domains, as well as a structured linker domain with variable sequence [39]. IGFBPs play an important role in insulin resistance in the course of PDAC [26]. IGFBP-3 is the main transporter of IGF proteins, followed by IGFBP-2 [40].

One of the better-known biomarkers for pancreatic cancer is the serum carbohydrate antigen CA 19-9, also known as Sialyl Lewis-A. It is expressed on pancreatic tumor cells and is used in cancer diagnosis with its monoclonal antibody [41]. The CA 19-9 level is considered useful in diagnosing PDAC and is also a sensitive indicator of disease recurrence after surgery [42,43]. Elevated CA 19-9 levels can manifest as early as 2 years before detecting PDAC, with 50% sensitivity and 99% specificity within 0–6 months prior to early PDAC cases [44]. Unfortunately, in asymptomatic patients, there is little evidence to support its clinical usefulness in screening [45,46,47,48]. In a study with 70,940 asymptomatic patients, the positive predictive value of CA 19-9 was only 0.9%, confirming the ineffectiveness of this diagnostic method for pancreatic cancer [48]. CA 19-9 is widely available and can be determined from a peripheral blood sample, representing prognostic value in PDAC [49,50,51]. Monitoring CA 19-9 levels after PDAC resection can predict the risk of relapse before CT/MRI. Approximately 60% of patients after resection showed a significant increase in CA 19-9 before recognizing relapse with imaging techniques. A 1.35-fold increase in CA 19-9 indicated relapse with 72% sensitivity and 62% specificity (85% positive predictive value (PPV) and 42% negative predictive value (NPV)). In contrast, a 2.45-fold increase in CA 19-9 level indicated relapse with 45% sensitivity and 85% specificity (90% PPV and 33% NPV). CA 19-9 levels greater than 130 U/mL indicate a nonresectable tumor. On the other hand, a decrease in concentration after surgery and normalization after treatment are associated with a favorable prognosis [43]. According to Polish guidelines, the CA 19-9 antigen is a known marker for PDAC, but it is not useful for early diagnosis or screening. However, determining this marker may be useful for assessing prognosis and predicting PDAC outcomes [52].

Some concerns related to CA 19-9 include its low sensitivity at an early stage of tumor development, normal values in individuals who do not produce this marker due to fucosyltransferase deficiency (leading to false-negative results), and clinical conditions in which its level is elevated, such as other diseases of the pancreas and bile ducts, including inflammation (leading to false-positive values) [53,54,55]. Additionally, elevated levels of CA 19-9 are present in diseases of the liver, thyroid, lungs, and spleen, as well as in diabetes and diseases of the reproductive system [56]. The marker’s levels can also increase in patients with cancer of the stomach, colon, lungs, thyroid, and bile ducts [57].

Approximately 10–20% of patients with chronic pancreatitis (CP) have inflammatory pancreatic abnormalities, such as a pancreatic mass, which can mimic pancreatic cancer on imaging [58,59]. In the course of CP, there are irreversible changes in the pancreatic parenchyma, which are closely correlated with long-term inflammation. These changes include fibrosis and atrophy of the gland, as well as disruption of the course of the pancreatic duct. A characteristic, but unfortunately difficult to differentiate, feature is the generalized enlargement of the pancreatic head in the form of a tumor, which is also typical of the location of pancreatic cancer [60]. The cumulative risk of PDAC increases, reaching 1.8% at 10 years and 4.0% at 20 years after the diagnosis of CP [61]. Due to the fact that CP is a risk factor for the PDAC development, it is extremely important to search for early cancer in the course of CP [62].

On the basis of the evidence presented above, the usefulness of determining IGF-1 and IGFBP-2 proteins, the IGF-1/IGFBP-2 ratio, and CA 19-9 in differentiating between PDAC and CP can be evaluated.

## 2. Material and Methods

The study enrolled 137 patients, including 89 with PDAC and 48 with CP. The PDAC group consisted of 42 women and 47 men with an average age of 65.43 (±10.11) years and an age range of 41–90 years. In the TNM classification, the majority of PDAC cases were in stage IV (43.8%), followed by stage IIIB (24.7%), and then stages IIA and IA (14.6% and 13.5%, respectively). The fewest PDAC cases were in stage IIIA (2.2%) and IIB (1.1%). According to the Cambridge classification, 30 CP patients had stage 3 (63%) and 18 had stage 2 (38%). The CP group consisted of 12 women and 36 men with an average age of 54.88 (±12.87) years and age range of 31–79 years (Table 1). The study was prospective and enrolled patients consecutively. The group of patients with PDAC and CP was expanded from a previous paper, including 40 patients with PDAC and 20 with CP, and the database was extended with 49 new cases of PDAC and 28 new cases of CP [33].

All subjects were tested for the levels of IGF-1 and IGFBP-2 using the ELISA method (Corgenix UK Ltd. R&D Systems, Lodz, Poland), as well as the level of CA 19-9 in serum. Additionally, the IGF-1/IGFBP-2 ratio was calculated. The study protocol was approved by the Bioethics Committee at the Medical University of Lodz (approval case number: RNN/108/15/KE).

Initial analysis was supported by the comparison of means of the indicators between the groups of PDAC and CP patients. The subsets of the sample were checked for normality using Shapiro–Wilk tests. Their results confirmed normality of all subsamples with *p* < 0.003. Drawing on that result, further comparisons were performed with the use of the Student *t*-test for the equality of means.

An attempt to assess the usefulness of the IGF-1, IGFBP-2, IGF-1/IGFBP-2 ratio, and CA 19-9 in the differentiation between PDAC and CP was undertaken. It was made in a formal way—with the use of logit and probit models. These models allow us to explain the behavior of dummy variables such as CP or PDAC existence with a set of determinants that include both continuous and dummy variables. In our case, the set of explanatory variables consisted of patients’ age and sex, as well as IGF-1, IGFBP-2, IGF-1/IGFBP-2 ratio, and CA 19-9 levels. The variables were used in different configurations. As a result, we obtained a class of models with the parameters that informed us about an impact of the changes in the values of given determinants on the value of probability that an individual developed CP or PDAC (expressed as logarithms of the odds ratio). The logit and probit models differ in the nature of assumptions that concern the distribution of probability of dependent variable; however, in our case, both models gave numerically equivalent results in each of analyzed iterations. The models were also free from the problem of unbalanced samples which might be an important factor that biases the estimates.

The efficiency of the models was assessed in order to study the usefulness of analyzed indicators in the diagnostic process. The efficiency was assessed by means of ROC curve construction. The ROC curves present the relationship between the sensitivity and specificity of the test statistic for different critical values. The efficiency of an indicator is measured by the size of the area under the ROC curve (AUROC), which expresses the probability that a given indicator will correctly subscribe randomly chosen individuals to their true categories. It is widely believed that a good indicator is characterized by an AUROC ranging between 0.8 and 0.9, whereas a very good indicator achieves an AUROC of 0.9–1. As the sample that we used consisted solely of the patients with PDAC and CP, by measuring the AUROC, we obtained information concerning the usefulness of analyzed indicators in differentiation between PDAC and CP. The results of these analyses for different possible model specifications are presented below.

## 3. Results

Below, we present the results of the analysis of the effectiveness of competing PDAC/CP indicators under different specifications. The analysis of individual indicators, including IGF-1, IGFBP-2, IGF-1/IGFBP-2 ratio, and CA 19-9, was gradually extended by allowing for combinations of indicators, as well as additional information concerning the age and sex of the patients. The results are presented in both graphical form, which supports their direct comparisons, and in tables, which include additional information concerning the uncertainty of the estimates given by their confidence intervals.

In the study group, CP was diagnosed on the basis of imaging materials, including abdominal CT, and the Rosemont and Cambridge criteria were used for the diagnosis of CP in endoscopic examinations such as EUS (endoscopic ultrasonography) and ERCP (endoscopic retrograde cholangiopancreatography). Difficulties in CP and PDAC differentiation concerned five patients (10.4%) suspected of a neoplastic tumor in the head of the pancreas in the course of CP.

An initial analysis consisted of the comparisons of the within-sample means of indicators. They proved that, in the case of IGF-1 (52.12 ± 33.13 ng/mL in PDAC vs. 74.23 ± 48.98 ng/mL in CP; *p* = 0.0053), IGFBP-2 (305.95 ± 194.58 ng/mL in PDAC vs. 485.43 ± 299 ng/mL in CP; *p* = 0.0002), and CA 19-9 (434.95 ± 419.98 U/mL in PDAC vs. 78.07 ± 182.36 U/mL in CP; *p* = 0.0000), we could reject the null hypothesis of the equality of means. In the PDAC and CP groups, the difference of means was not confirmed in case of the IGF-1/IGFBP-2 ratio (0.213 ± 0.14 in PDAC vs. 0.277 ± 0.33 in CP; *p* = 0.1914), which might undermine the usefulness of that indicator for the purpose of differentiation between the CP and PDAC.

**Figure 1:**
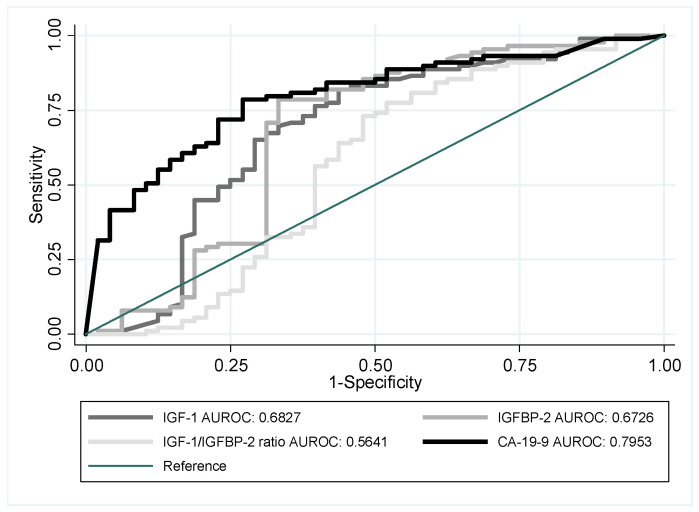
ROC curves of individual indicators: IGF-1, IGFBP-2, IGF-1/IGFBP-2 ratio, and CA 19-9 used for the purpose of PDAC and CP differentiation.

Figure 1 presents the efficiency of individual analyzed indicators in PDAC/CP differentiation. It is evident that the IGF-1, IGFBP-2, and IGF-1/IGFBP-2 ratio did not behave better than CA 19-9, which is currently the basic indicator used in PDAC diagnosis. Each of these indicators quickly lost specificity, even for low sensitivity levels. In each of these cases, the AUROC ranged below 0.7, being strictly lower than the AUROC of 0.7953 that characterized the CA 19-9 indicator. Even though the result for CA 19-9 was insignificantly different from 0.8, which is a limiting value for good indicators, the result was not robust when considering the uncertainty of the ROC curve estimate. The 95% confidence interval limited the possible AUROC of CA 19-9 to the level of 0.719. 

The performance of analyzed indicators in the differentiation between PDAC and CP did not change significantly when additional information concerning whether the patients suffered from DM was included. As shown in Table 2, the most noticeable increase in the value of AUROC was observed in the case of an indicator which combined information about the DM and IGF-1 level; however, even in that case, it did not improve the result by more than 0.014. In case of the improvement of other indicators, they were either of the fourth order of magnitude or nonexistent. This observation led us to the conclusion that information concerning the occurrence of DM among patients did not allow for better differentiation between CP and PDAC. In the subsequent analyses, we refrained from including DM as a possible determinant of any of these diseases.

**Figure 2:**
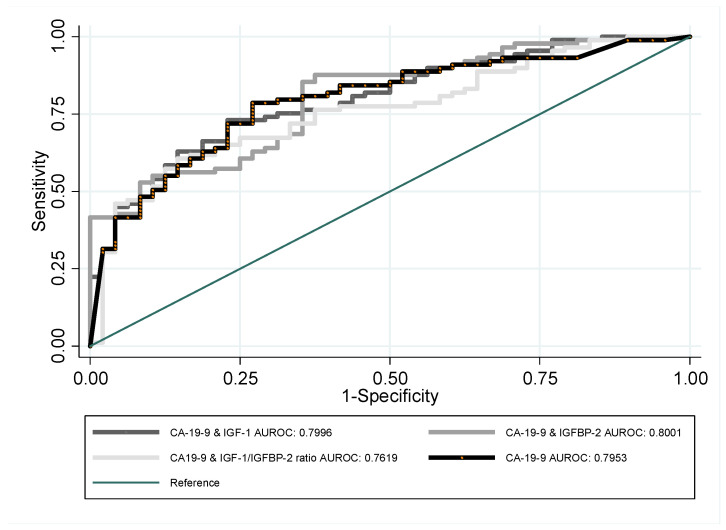
ROC curves of CA 19-9 together with IGF-1, IGFBP-2, or IGF-1/IGFBP-2 ratio, used for the purpose of PDAC and CP differentiation.

Due to the promising performance of CA 19-9, we decided to improve its efficiency in the differentiation of PDAC and CP by combining it with the other analyzed indicators. The ROC curves for a new set of indicators are presented in Figure 2. Such an approach resulted in much better performance of the indicators, particularly CA 19-9 + IGFBP-2. For some sensitivity levels, it outperformed the individual CA 19-9 result; however, the overall impact was not particularly impressive, as the AUROC of combined indicators only increased to 0.8 and still ranged far below 0.8 when uncertainty was considered. 

**Figure 3:**
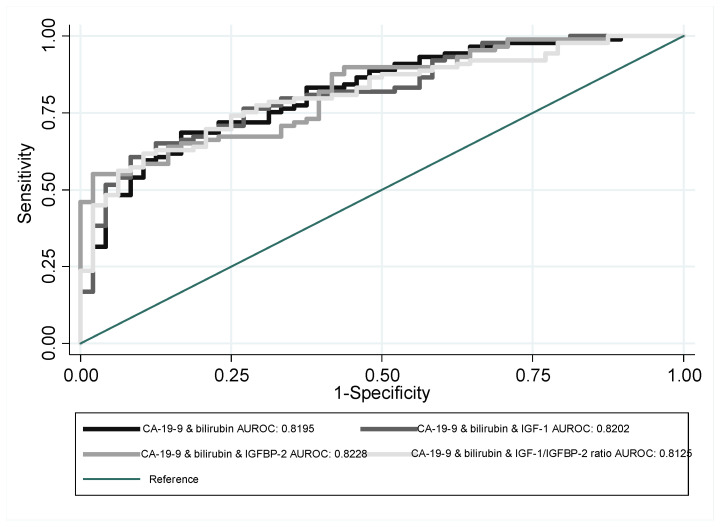
ROC curves of CA 19-9 and bilirubin together with IGF-1, IGFBP-2, or IGF-1/IGFBP-2 ratio, used for the purpose of PDAC and CP differentiation.

**Figure 4:**
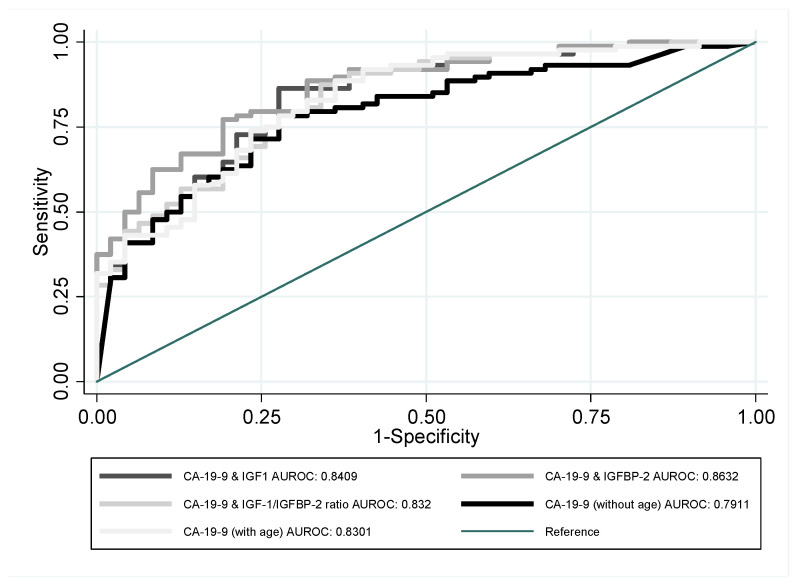
ROC curves of patients’ age and CA 19-9 acting together with IGF-1, IGFBP-2, or IGF-1/IGFBP-2 ratio, used for the purpose of PDAC and CP differentiation.

Substantially greater AUROC growth was achieved when bilirubin levels and patients’ age were included as indicators. Bilirubin combined with CA 19-9 increased the efficiency of this indicator by approximately 0.02. Similar results were obtained when CA 19-9 and bilirubin levels were used with the other indicators. Individual indicators combined with age (where age was treated as a factor which increased the probability of PDAC occurrence) still underperformed compared with CA 19-9 (Table 2); the results for the case which combined patients’ age and CA 19-9 with the other indicators, especially IGFBP-2, performed much better. In the latter case, the AUROC was equal to 0.8632, and its 95% confidence interval held above the 0.8 limit. The resulting indicator’s ROC curve outperformed the other indicators over the majority of specificity values (Figure 3 and Figure 4). BMI was also tested as one of possible differentiating factors; however, we refrained from publishing the results of these analyses as they resulted in significant sample curtail, making the results incomparable. Nevertheless, no signs of indicator performance improvement were witnessed.

In the final attempt, we also extended an indicator by including information about the patients’ sex (considering that men were more frequently diagnosed with CP); however, in this case, the increase was not significant. Once again, the indicator which included CA 19-9 and IGFBP-2 outperformed the other indicators, reaching an AUROC of 0.8711. The result was also robust if the bilirubin level was taken into account (Table 2).

According to the existing evidence, the CA 19-9 marker turned out to be more efficient when used for the purpose of differentiating developed PDACs, which ultimately constituted its major weakness. As such, the efficiency of individual indicators might differ when the maturity of neoplastic changes is taken into account. In order to verify this, we divided our sample of PDAC patients into two groups depending on the TNM status of their changes. Patients with the changes of ranks I and II were assessed as patients with early PDAC. Thos group consisted of 45 persons (including 25 men) with a mean age of 65.02 years. The second group of patients with advanced PDAC consisted of persons of TNM ranks III and IV. This group consisted of 44 persons (including 22 men) with an average age of 65.84 years. These two subsamples were compared with an original group of CP patients which consisted of 53 patients, as we concluded that further divisions might give rise to the problem of unbalanced samples and, thus, affect the efficiency of logit/probit estimators. 

The mean values of IGF-1, IGFBP-2, and IGF-1/IGFBP-2 ratio did not differ significantly in the groups with early and advanced PDAC (IGF-1: 51.79 ng/mL vs. 51.25 ng/mL, *p* = 0.9382; IGFBP-2: 296.16 ng/mL vs. 307.15 ng/mL, *p* = 0.7872; IGF-1/IGFBP-2 ratio: 0.21 vs. 0.22, *p* = 0.7778). Only in the case of CA 19-9 was the difference considerable and statistically significant, 306.46 U/mL vs. 534.78 U/mL (*p* = 0.0096), in line with the existing scientific record. For between-group differences with the CP sample, in the case of early PDAC, we found evidence of means differences for IGF-1 (51.79 ng/mL in early PDAC vs. 79.16 ng/mL in CP; *p* = 0.0019), IGFBP-2 (296.16 ng/mL in early PDAC vs. 505.21 ng/mL in CP; *p* = 0.0001), and CA 19-9 (306.46 U/mL in early PDAC vs. 80.21 U/mL in CP; *p* = 0.0006). When the group of advanced PDACs was compared with the group of CP patients, statistically significant differences of means were observed for IGF-1 (51.25 ng/mL in advanced PDAC vs. 79.16 ng/mL in CP; *p* = 0.0036), IGFBP-2 (307.15 ng/mL in advanced PDAC vs. 505.21 ng/mL in CP; *p* = 0.0004), and CA 19-9 (306.46 U/mL in advanced PDAC vs. 80.21 U/mL in CP; *p* = 0.0000).

Having split the PDAC sample, further analyses were performed by means of AUROC computation for each specification of the diagnostic indicator that were used in the full sample in order to assess how their efficiency in PDAC vs. CP differentiation varies depending on the maturity of neoplastic changes. The results of these analyses are presented in Table 3.

The general conclusion resulting from the performed estimations points toward a better efficiency of CA 19-9 in the differentiation between advanced PDAC and CP. Indicators which were constructed around CA 19-9 reported an AUROC which was, on average, 0.1 higher in the case when used to differentiate between advanced PDAC and CP compared to the situation in which early PDAC was differentiated from CP. Indicators which used data concerning patients’ age, sex, bilirubin, and CA 19-9 levels demonstrated AUROCs higher than 0.9, characterized as very good indicators. The best among them was an indicator which used data on patients’ age, sex, bilirubin, CA 19-9, and IGFBP-2 levels, which was characterized by an AUROC of 0.9338. The same indicator was also the best in the differentiation of early-stage PDAC from CP; however, in that case, the AUROC reached only the level of 0.8433.

Patients’ age is considered to be another important factor facilitating differentiation of advanced PDAC from CP. The average age of patients with advanced PDAC was almost equal to that with early PDAC and CP; however, it was much more concentrated around the average as its standard deviation was equal to 8.51 years compared to 11.59 and 12.73, respectively. In cases when indicators consisting of information on patients’ age and their IGF-1 or IGFBP-2 serum levels were used to differentiate between advanced PDAC and CP, the AUROCs reached the level of 0.912 and 0.916, respectively, only 0.08–0.04 worse than in the case when the most efficient but much more complicated indicator based on patients’ age, sex, CA 19-9, and IGFBP-2 levels was used.

Only four indicators out of 33 proved to be better at differentiation of early PDAC from CP than advanced PDAC from CP. Two of them used solely the data concerning the IGF-1 and IGFBP-2 serum levels, whereas the other two combined the IGFBP-2 and IGF-1/IGFBP-2 ratio levels with the data concerning DM. The overall benefits were, however, merely symbolic as the difference in performance between samples was no higher than 0.044.

## 4. Discussion

This study showed that the serum levels of IGF-1 were significantly elevated in CP patients compared to the PDAC group (*p* = 0.0053). However, the literature on IGF-1 in pancreatic cancer is inconsistent, with some studies confirming high levels in both CP and PDAC, while others do not [63,64]. The changes in IGF-1 concentration observed in pancreatic cancer patients may be attributed to differences in tumor stroma composition and secreted proteins of the IGF axis.

Hyperinsulinemia, which is common in the course of PDAC, can reduce the level of IGF-1 [65]. Insulin stimulates the synthesis of IGF-1 in the liver and inhibits the hepatic synthesis of IGFBP-1, which increases the level of IGF-1 in the serum [66]. Other studies have also shown a relationship of the amount of adipose tissue, peripheral insulin resistance, and the level of IGF-1, as adipose tissue is one of the sources of IGF-1, and insulin resistance accompanying obesity increases the level of this protein [67,68].

Increased IGF-1 levels were found only in six out of 30 patients with PDAC in Basso et al.’s study. The lower level of IGF-1 in PDAC could be explained by the impact of GH stimulation, liver production, or tumor synthesis [63]. Meggiato et al. also found increased IGF-1 serum levels in only 10% of patients with pancreatic cancer, and their analysis showed no differences in IGF-1 according to tumor stage or size [64]. In contrast, our study found no significant differences in IGF-1 levels according to the clinical stage of pancreatic cancer.

In our study, we found that the serum level of IGFBP-2 was significantly elevated in CP patients compared to the PDAC group (*p* = 0.0002). Previous studies have shown that increased levels of IGFBP-2 are associated with early PanIN lesions and worse patient survival, suggesting its role as a key prognostic marker in the progression of PDAC [65]. The role of IGF and IGFBP proteins in the stroma of the pancreas is still under analysis [26]. Increased expression of IGFBP-2 in PDAC affects the invasion of cancer cells and the formation of metastases by influencing the NF-κB-dependent epithelial–mesenchymal transition [69]. Dong et al. found that tumor IGFBP-2 expression is related to plasma IGFBP-2 levels, which are also correlated with overall survival. They also observed a significant association between IGFBP-2 levels and malnutrition and muscle wasting in PDAC [70].

Regarding the IGF-1/IGFBP-2 ratio, our study and a previous study were the only ones to test it in CP and PDAC patients. We found that the IGF-1/IGFBP-2 ratio was a very good marker to confirm PDAC cases compared to control healthy subjects. However, in our current study, with a larger sample group including CP cases, this marker lost its sensitivity in differentiating CP from PDAC. 

One of the results of this study was a significant increase in the level of CA 19-9 in the PDAC group compared to the CP group (*p* = 0.0000). However, serum CA 19-9 is not recommended as a standalone biomarker for early diagnosis of PDAC due to its lack of sensitivity and specificity. Currently, there is still a need for more sensitive and specific biomarkers for pancreatic cancer [71]. False negatives in CA 19-9-negative patients and false positives in the presence of other conditions such as pancreatitis or cholangitis can impair the potential role of CA 19-9. Nevertheless, CA 19-9 testing may be useful in detecting pancreatic cancer recurrence [51]. Hong et al. demonstrated that a high level of CA 19-9 (cutoff value > 70 U/mL) was predictive of early recurrence of early PDAC. In their study of 407 patients, multivariate analysis showed that CA 19-9 level > 70 U/mL (*p* = 0.006), tumor size > 2.85 cm (*p* = 0.004), poor differentiation (*p* = 0.008), and non-adjuvant chemotherapy (*p* = 0.025) were significant risk factors for early pancreatic cancer [49]. In van Manen et al.’s study, researchers investigated whether carcinoembryonic antigen (CEA) and CA 19-9 markers could predict the stage of PDAC in a total of 375 patients. The optimal cutoff values for predicting advanced PDAC were found to be 7.0 ng/mL for CEA and 305.0 U/mL for CA 19-9, resulting in positive predictive values of 83.3%, 73.6%, and 91.4% for CEA, CA 19-9, and their combination, respectively. CEA and CA 19-9 were found to be independent predictors of advanced PDAC, with odds ratios of 4.21 (95% CI: 1.85–9.56; *p* = 0.001) for CEA and 2.58 (95% CI: 1.30–5.14; *p* = 0.007) for CA 19-9 [72]. Currently, CA 19-9 is the only marker used for the diagnosis and differentiation of PDAC and to assess the progression and treatment of pancreatic cancer [44].

One of the conclusions of this study was that IGF-1, IGFBP-2, and the IGF-1/IGFBP-2 ratio did not perform better than CA 19-9 in differentiating between PDAC and CP. Each of these markers quickly lost specificity as sensitivity increased, confirming the key role of CA 19-9 as a basic indicator in PDAC diagnosis. The CA 19-9 AUROC was the highest among other parameters tested, measuring 0.7953. In contrast, the AUROC of each of the other markers ranged below 0.7, which was significantly lower. Additionally, it is noteworthy that CA 19-9 was not significantly different from 0.8, which is the limit for good indicators. Other researchers are constantly seeking the perfect panel of markers for early detection and differentiation of PDAC from CP. Mehta et al. investigated a panel of four biomarkers (S100A2, S100A4, CA-125, and CA 19-9) that showed significant diagnostic potential (AUROC = 0.913). The biomarkers (S100A4, CA-125, and CA 19-9) correlated with poor overall survival (*p* < 0.05). Pancreatic cancer patients with abnormal levels of two or more of the presented markers had significantly shorter survival than patients with an abnormal result of ≤1 marker (*p* < 0.05) [73]. In our further analysis, we aimed to improve the efficiency of CA 19-9 in differentiating between CP and PDAC by combining it with other analyzed indicators: IGF-1, IGFBP-2, and the IGF-1/IGFBP-2 ratio. This approach resulted in better performance of these indicators, especially the CA 19-9 + IGFBP-2 combination. When we added patients’ age to the calculations, we achieved an increase in AUROC. However, the combination of IGF-1 and IGFBP-2 with age still performed worse in differentiating between PDAC and CP compared to CA 19-9 alone. The results for patients’ age, CA 19-9, and IGFBP-2 taken together performed much better, with an AUROC of 0.8632, and its 95% confidence interval holding above the 0.8 limit.

In a similar study, Hrabak et al. used a combination of indicators including IGF-1, IGFBP-2, and CA 19-9. They analyzed 28 PDAC and 47 CP for 58 biomarkers and compared their performance in detecting PDAC to the levels of CA 19-9. The panel defined by random forest (RF) analysis, including CA 19-9, AAT, IGFBP-2, albumin, ALP, Reg3A, and HSP27, outperformed CA 19-9 alone in discriminating PDAC from DM (AUROC 0.92 vs. 0.82). The panel of CA 19-9, S100P-pl, AAT, albumin, adiponectin, IGF-1, MMP7, and S100A11 identified PDAC better than CA 19-9 alone (AUROC 0.91 vs. 0.80). However, the panel defined by logistic regression analysis, consisting of prealbumin, IGFBP-2, DJ-1, MIC-1, and CA72-4, discriminated PDAC from DM worse than CA 19-9 (AUROC 0.80 vs. 0.82). The panel of IGF-1, S100A11, and Reg1alfa outperformed CA 19-9 in discriminating PDAC from CP (AUROC 0.76 vs. 0.75) [74]. This study confirms that the combination of markers is more useful in diagnosing patients with pancreatic cancer than the determination of a single CA 19-9 marker. However, the AUROC in that case was still much lower compared to our results. We also found that adding information concerning patients’ sex did not significantly increase the performance. Only an indicator that included gender, CA 19-9, and IGFBP-2 outperformed the other indicators, reaching an AUROC of 0.8711, which reliably increased the sensitivity in detecting PDAC. On the basis of previous studies, it was thought that the IGF-1/IGFBP-2 ratio could differentiate patients with pancreatic cancer from the control group. However, our present results confirm that the IGF-1/IGFBP-2 ratio can only differentiate general pancreatic diseases from the group of healthy patients.

Our study had several limitations, including a relatively small sample size of patients with PDAC and CP, which may have resulted in slightly different results in the general population. Another limitation was the lack of control group, whose protein level results could be used in future studies to assess the diagnostic utility of the markers. Moreover, the deficiency in determining the CA 19-9 level was affected by the advanced stage of PDAC and a small number of early pancreatic cancers. The study’s power was also limited by the small number of patients diagnosed with early PDAC, and results may have been influenced by the inadequate number of patients with new-onset diabetes.

The IGF system has been implicated in pancreatic cancer oncogenesis due to its role in cancer cell proliferation and metastasis formation. However, the impact of IGF axis proteins on pancreatic stromal cells and the development of pancreatic diabetes requires further investigation. Our study confirmed that CA 19-9 is a reliable biomarker for differentiating PDAC from CP patients, but we also demonstrated the diagnostic potential of an innovative panel that includes age, CA 19-9, and IGFBP-2. This panel outperformed CA 19-9 alone, with an AUROC of 0.8632 and a 95% confidence interval that remained above the 0.8 threshold in distinguishing PDAC from CP cases.

## Figures and Tables

**Table 1:** The main clinical features of the PDAC and CP group.

	89 PDAC	48 CP
Sex	42 women and 47 men	12 women and 36 men
Age	65.43 (±10.11)	54.88 (±12.87)
BMI kg/m^2^	23.8 ± 5.0	23.8 ± 0.6
DM	29	18
Bilirubin level (norm: 0.8–1.2 mg/dL)	6.13 ± 8.98	1.15 ± 1.39

**Table 2:** Summary of performed ROC curve analyses—PDAC vs. CP differentiation.

Indicator	Obs	AUROC	Std. Err.	95% Conf. Interval	
IGF-1	137	0.6827	0.0532	0.57834	0.78707
IGFBP-2	137	0.6726	0.0559	0.56298	0.78229
IGF-1/IGFBP-2 ratio	137	0.5641	0.0587	0.44904	0.67924
DM + IGF-1	137	0.6963	0.0511	0.59615	0.7964
DM + IGFBP-2	137	0.6723	0.056	0.56258	0.78199
DM + IGF-1/IGFBP-2 ratio	137	0.4649	0.0573	0.35267	0.5771
DM + CA-19-9	137	0.7958	0.0385	0.72022	0.8713
CA-19-9	137	0.7953	0.0389	0.71904	0.87155
CA-19-9 + IGF-1	137	0.7996	0.0377	0.72565	0.8736
CA-19-9 + IGFBP-2	137	0.8001	0.0378	0.72605	0.87414
CA-19-9 + IGF-1/IGFBP-2 ratio	137	0.7619	0.0413	0.68103	0.84284
CA-19-9 + bilirubin	137	0.8195	0.0364	0.74821	0.89084
CA-19-9 + bilirubin + IGF-1	137	0.8202	0.0359	0.74995	0.8905
CA-19-9 + bilirubin + IGFBP-2	137	0.8228	0.0346	0.75489	0.89071
CA-19-9 + bilirubin + IGF-1/IGFBP-2 ratio	137	0.8125	0.0362	0.74162	0.88338
Age + IGF-1	135	0.7747	0.0432	0.68998	0.85935
Age + IGFBP-2	135	0.817	0.0394	0.73965	0.89429
Age + IGF-1/IGFBP-2 ratio	135	0.7672	0.0449	0.67924	0.85509
Age + CA-19-9	135	0.8301	0.0367	0.75829	0.90201
Age + CA-19-9 + IGF-1	135	0.8409	0.0352	0.77189	0.90993
Age + CA-19-9 + IGFBP-2	135	0.8632	0.0314	0.80161	0.9247
Age + CA-19-9 + IGF-1/IGFBP-2 ratio	135	0.832	0.0362	0.76108	0.90285
Age + sex + IGF-1	135	0.7923	0.0428	0.70845	0.87617
Age + sex + IGFBP-2	135	0.8332	0.0381	0.75845	0.90789
Age + sex + IGF-1/IGFBP-2 ratio	135	0.7841	0.0447	0.69646	0.87172
Age + sex + CA-19-9	135	0.8347	0.0361	0.76399	0.9055
Age + sex + CA-19-9 + IGF-1	135	0.8467	0.0345	0.77912	0.91431
Age + sex + CA-19-9 + IGFBP-2	135	0.8711	0.0309	0.81052	0.93174
Age + sex + CA-19-9 + IGF-1/IGFBP-2 ratio	135	0.8433	0.0349	0.77489	0.91176
Age + sex + bilirubin + CA-19-9	135	0.8501	0.034	0.78347	0.91672
Age + sex + bilirubin + CA-19-9 + IGF-1	135	0.8622	0.0319	0.79973	0.92464
Age + sex + bilirubin + CA-19-9 + IGFBP-2	135	0.8832	0.0281	0.82808	0.93836
Age + sex + bilirubin + CA-19-9 + IGF-1/IGFBP-2 ratio	135	0.8648	0.0309	0.8042	0.92549

**Table 3:** Summary of performed ROC curve analyses—early and advanced PDAC vs. CP.

Indicator	CP vs. PDAC (TNM = I & II)					CP vs. PDAC (TNM = III & IV)				
	Obs	AUROC	Std. Err.	95% CI		Obs	AUROC	Std. Err.	95% CI	
IGF-1	93	0.6704	0.0581	0.55649	0.78425	92	0.6953	0.0576	0.58243	0.80819
IGFBP-2	93	0.6822	0.0588	0.56695	0.7974	92	0.6629	0.0596	0.54601	0.77974
IGF-1/IGFBP-2 ratio	93	0.5801	0.0627	0.45722	0.70296	92	0.5478	0.0619	0.42658	0.66907
DM + IGF-1	93	0.6894	0.0559	0.57975	0.79895	92	0.6986	0.0575	0.58598	0.81127
DM + IGFBP-2	93	0.7056	0.055	0.59783	0.81328	92	0.6615	0.0602	0.5434	0.77952
DM + IGF-1/IGFBP-2 ratio	93	0.4917	0.0617	0.37068	0.61265	92	0.4607	0.062	0.33926	0.58215
DM + CA-19-9	93	0.735	0.0538	0.62952	0.84038	92	0.866	0.0396	0.78845	0.94356
CA-19-9	93	0.7294	0.0523	0.62697	0.83183	92	0.8627	0.0405	0.78325	0.94213
CA-19-9 + IGF-1	93	0.7315	0.052	0.62965	0.83331	92	0.866	0.0388	0.78987	0.94214
CA-19-9 + IGFBP-2	93	0.7546	0.0499	0.6568	0.85246	92	0.8532	0.0389	0.77692	0.92952
CA-19-9 + IGF-1/IGFBP-2 ratio	93	0.6685	0.0564	0.55791	0.77913	92	0.8627	0.0403	0.78371	0.94167
CA-19-9 + bilirubin	93	0.725	0.0522	0.62261	0.82739	92	0.9138	0.0279	0.85912	0.96853
CA-19-9 + bilirubin + IGF-1	93	0.7505	0.0504	0.6516	0.84933	92	0.9029	0.0319	0.84038	0.96549
CA-19-9 + bilirubin + IGFBP-2	93	0.7648	0.0487	0.66937	0.86026	92	0.91	0.029	0.85317	0.9669
CA-19-9 + bilirubin + IGF-1/IGFBP-2 ratio	93	0.7255	0.0522	0.6231	0.82782	92	0.9063	0.0316	0.84436	0.96814
Age + IGF-1	91	0.7814	0.048	0.6873	0.87556	91	0.912	0.0288	0.85564	0.96834
Age + IGFBP-2	91	0.8071	0.0453	0.71824	0.89588	91	0.9159	0.0274	0.86208	0.96965
Age + IGF-1/IGFBP-2 ratio	91	0.7505	0.0512	0.65019	0.85078	91	0.9038	0.0298	0.84532	0.96223
Age + CA-19-9	91	0.7621	0.0496	0.66483	0.85935	91	0.903	0.0302	0.84391	0.96218
Age + CA-19-9 + IGF-1	91	0.7853	0.0475	0.69216	0.87844	91	0.8138	0.0444	0.72674	0.90092
Age + CA-19-9 + IGFBP-2	91	0.8201	0.0437	0.73441	0.90582	91	0.8288	0.0428	0.745	0.91264
Age + CA-19-9 + IGF-1/IGFBP-2 ratio	91	0.7592	0.0497	0.66173	0.85664	91	0.7834	0.0488	0.68767	0.87906
Age + sex + IGF-1	91	0.7877	0.0473	0.69496	0.88048	91	0.8308	0.0421	0.74822	0.91329
Age + sex + IGFBP-2	91	0.8269	0.0438	0.74113	0.91264	91	0.8482	0.04	0.76982	0.9265
Age + sex + IGF-1/IGFBP-2 ratio	91	0.7722	0.0495	0.67528	0.8692	91	0.8061	0.0466	0.71478	0.89741
Age + sex + CA-19-9	91	0.779	0.0484	0.68423	0.8738	91	0.9021	0.0302	0.84296	0.9612
Age + sex + CA-19-9 + IGF-1	91	0.7993	0.0457	0.7097	0.88894	91	0.9125	0.0286	0.85651	0.96844
Age + sex + CA-19-9 + IGFBP-2	91	0.837	0.0424	0.75396	0.92012	91	0.9202	0.0264	0.86848	0.97194
Age + sex + CA-19-9 + IGF-1/IGFBP-2 ratio	91	0.7838	0.0476	0.69065	0.87705	91	0.9062	0.0294	0.8486	0.96378
Age + sex + bilirubin + CA-19-9	91	0.7969	0.046	0.7068	0.88701	91	0.927	0.0249	0.87824	0.97573
Age + sex + bilirubin + CA-19-9 + IGF-1	91	0.8133	0.0441	0.72697	0.89972	91	0.9318	0.0237	0.88531	0.97833
Age + sex + bilirubin + CA-19-9 + IGFBP-2	91	0.8433	0.0408	0.76344	0.92321	91	0.9338	0.0236	0.88757	0.97994
Age + sex + bilirubin + CA-19-9 + IGF-1/IGFBP-2 ratio	91	0.7998	0.0456	0.71035	0.88927	91	0.9275	0.0248	0.87888	0.97605

## Data Availability

The data presented in this study are available on request from the corresponding author.

## References

[1] Allemani C., Matsuda T., Di Carlo V., Harewood R., Matz M., Nikšić M., Bonaventure A., Valkov M., Johnson C.J., Estève J. (2018). Global surveillance of trends in cancer survival 2000–14 (CONCORD-3): Analysis of individual records for 37 513 025 patients diagnosed with one of 18 cancers from 322 population-based registries in 71 countries. Lancet.

[2] Liot S., Balas J., Aubert A., Prigent L., Mercier-Gouy P., Verrier B., Bertolino P., Hennino A., Valcourt U., Lambert E. (2021). Stroma Involvement in Pancreatic Ductal Adenocarcinoma: An Overview Focusing on Extracellular Matrix Proteins. Front. Immunol..

[3] Pandol S., Edderkaoui M., Gukovsky I., Lugea A., Gukovskaya A. (2009). Desmoplasia of Pancreatic Ductal Adenocarcinoma. Clin. Gastroenterol. Hepatol..

[4] Omary M.B., Lugea A., Lowe A.W., Pandol S.J. (2007). The pancreatic stellate cell: A star on the rise in pancreatic diseases. J. Clin. Investig..

[5] Korc M. (2007). Pancreatic cancer–associated stroma production. Am. J. Surg..

[6] Chu G.C., Kimmelman A.C., Hezel A.F., DePinho R.A. (2007). Stromal biology of pancreatic cancer. J. Cell. Biochem..

[7] Cotterill A.M., Holly J.M.P., Wass J.A.H. (1993). The regulation of insulin-like growth factor binding protein (IGFBP)-1 during prolonged fasting. Clin. Endocrinol..

[8] Mullis P.E., Patel M.S., Brlckell P.M., Hindmarsh P.C., Brook C.G.D. (1991). Growth characteristics and response to growth hormone therapy in patients with hypochondroplasia: Genetic linkage of the insulin-like growth factor I gene at chromosome 12q23 to the disease in a subgroup of these patients. Clin. Endocrinol..

[9] Grimberg A., DiVall S.A., Polychronakos C., Allen D.B., Cohen L.E., Quintos J.B., Rossi W.C., Feudtner C., Murad M.H., Drug and Therapeutics Committee and Ethics Committee of the Pediatric Endocrine Society (2016). Guidelines for Growth Hormone and Insulin-Like Growth Factor-I Treatment in Children and Adolescents: Growth Hormone Deficiency, Idiopathic Short Stature, and Primary Insulin-Like Growth Factor-I Deficiency. Horm. Res. Paediatr..

[10] Gubbi S., Quipildor G.F., Barzilai N., Huffman D.M., Milman S. (2018). IGF-1: The Jekyll & Hyde of the aging brain. J. Mol. Endocrinol..

[11] Pollak M. (2008). Insulin and insulin-like growth factor signalling in neoplasia. Nat. Rev. Cancer.

[12] Clemmons D.R. (2004). The relative roles of growth hormone and IGF-1 in controlling insulin sensitivity. J. Clin. Investig..

[13] Yakar S., Sun H., Zhao H., Pennisi P., Toyoshima Y., Setser J., Stannard B., Scavo L., Leroith D. (2005). Metabolic effects of IGF-I deficiency: Lessons from mouse models. Pediatr. Endocrinol. Rev..

[14] Yang J., Waldron R.T., Su H.-Y., Moro A., Chang H.-H., Eibl G., Ferreri K., Kandeel F.R., Lugea A., Li L. (2016). Insulin promotes proliferation and fibrosing responses in activated pancreatic stellate cells. Am. J. Physiol. Liver Physiol..

[15] E Forbes B. (2016). Two years in IGF research. Growth Horm. IGF Res..

[16] Rajah R., Katz L., Nunn S., Solberg P., Beers T., Cohen P. (1995). Insulin-like growth factor binding protein (IGFBP) proteases: Functional regulators of cell growth. Prog. Growth Factor Res..

[17] Sun L., Zhang X., Song Q., Liu L., Forbes E., Tian W., Zhang Z., Kang Y., Wang H., Fleming J.B. (2020). IGFBP2 promotes tumor progression by inducing alternative polarization of macrophages in pancreatic ductal adenocarcinoma through the STAT3 pathway. Cancer Lett..

[18] Renehan A.G., Zwahlen M., Minder C., O’Dwyer S.T., Shalet S.M., Egger M. (2004). Insulin-like growth factor (IGF)-I, IGF binding protein-3, and cancer risk: Systematic review and meta-regression analysis. Lancet.

[19] Yu H., Rohan T. (2000). Role of the Insulin-Like Growth Factor Family in Cancer Development and Progression. Gynecol. Oncol..

[20] Zang G., Sandberg M., Carlsson P.O., Welsh N., Jansson L., Barbu A. (2015). Activated pancreatic stellate cells can impair pancreatic islet function in mice. Upsala J. Med. Sci..

[21] Tommelein J., De Vlieghere E., Verset L., Melsens E., Leenders J., Descamps B., De Wever O. (2018). Radiotherapy-activated cancer-associated fibroblasts promote tumor progression through paracrine IGF1R activation. Cancer Res..

[22] Siddle K. (2011). Signalling by insulin and IGF receptors: Supporting acts and new players. J. Mol. Endocrinol..

[23] Hirakawa T., Yashiro M., Doi Y., Kinoshita H., Morisaki T., Fukuoka T., Hirakawa K. (2016). Pancreatic fibroblasts stimulate the motility of pancreatic cancer cells through IGF1/IGF1R signaling under hypoxia. PLoS ONE.

[24] Manoukian P., Bijlsma M., van Laarhoven H. (2021). The Cellular Origins of Cancer-Associated Fibroblasts and Their Opposing Contributions to Pancreatic Cancer Growth. Front. Cell. Dev. Biol..

[25] Sternlicht M.D., Werb Z. (2001). How Matrix Metalloproteinases Regulate Cell Behavior. Annu. Rev. Cell. Dev. Biol..

[26] Thomas D., Radhakrishnan P. (2020). Role of Tumor and Stroma-Derived IGF/IGFBPs in Pancreatic Cancer. Cancers.

[27] Tape C.J., Ling S., Dimitriadi M., McMahon K.M., Worboys J.D., Leong H.S., Jørgensen C. (2016). Oncogenic KRAS regulates tumor cell signaling via stromal reciprocation. Cell.

[28] Mutgan A.C., Besikcioglu H.E., Wang S., Friess H., Ceyhan G.O., Demir I.E. (2018). Insulin/IGF-driven cancer cell-stroma crosstalk as a novel therapeutic target in pancreatic cancer. Mol. Cancer.

[29] Cai Q., Dozmorov M., Oh Y. (2020). IGFBP-3/IGFBP-3 Receptor System as an Anti-Tumor and Anti-Metastatic Signaling in Cancer. Cells.

[30] Jiang Q., Lou K., Hou L., Lu Y., Sun L., Tan S.C., Low T.Y., Kord-Varkaneh H., Pang S. (2020). The effect of resistance training on serum insulin-like growth factor 1(IGF-1): A systematic review and meta-analysis. Complement. Ther. Med..

[31] Wlodarczyk B., Gasiorowska A., Borkowska A., Malecka-Panas E. (2017). Evaluation of insulin-like growth factor (IGF-1) and retinol binding protein (RBP-4) levels in patients with newly diagnosed pancreatic adenocarcinoma (PDAC). Pancreatology.

[32] Wlodarczyk B., Borkowska A., Włodarczyk P., Malecka-Panas E., Gasiorowska A. (2019). Serum Levels of Insulin-like Growth Factor 1 and Insulin-like Growth Factor–binding Protein 2 as a Novel Biomarker in the Detection of Pancreatic Adenocarcinoma. J. Clin. Gastroenterol..

[33] Włodarczyk B., Borkowska A., Włodarczyk P., Małecka-Panas E., Gąsiorowska A. (2021). Insulin-like growth factor 1 and insulin-like growth factor binding protein 2 serum levels as potential biomarkers in differential diagnosis between chronic pancreatitis and pancreatic adenocarcinoma in reference to pancreatic diabetes. Gastroenterol. Rev..

[34] Rhim A.D., Oberstein P.E., Thomas D.H., Mirek E.T., Palermo C.F., Sastra S.A., Stanger B.Z. (2014). Stromal elements act to restrain, rather than support, pancreatic ductal adenocarcinoma. Cancer Cell.

[35] Ceyhan G.O., Schäfer K.-H., Kerscher A.G., Rauch U., Demir I.E., Kadihasanoglu M., Böhm C., Müller M.W., Büchler M.W., Giese N.A. (2010). Nerve Growth Factor and Artemin Are Paracrine Mediators of Pancreatic Neuropathy in Pancreatic Adenocarcinoma. Ann. Surg..

[36] Kikuta K., Masamune A., Hamada S., Takikawa T., Nakano E., Shimosegawa T. (2013). Pancreatic stellate cells reduce insulin expression and induce apoptosis in pancreatic β-cells. Biochem. Biophys. Res. Commun..

[37] Azzarelli R., Hurley C., Sznurkowska M., Rulands S., Hardwick L., Gamper I., Ali F.T., McCracken L., Hindley C., McDuff F. (2017). Multi-site Neurogenin3 Phosphorylation Controls Pancreatic Endocrine Differentiation. Dev. Cell..

[38] Ianza A., Sirico M., Bernocchi O., Generali D. (2021). Role of the IGF-1 Axis in Overcoming Resistance in Breast Cancer. Front. Cell Dev. Biol..

[39] Firth S.M., Baxter R. (2002). Cellular Actions of the Insulin-Like Growth Factor Binding Proteins. Endocr. Rev..

[40] Kendrick Z.W., Firpo M.A., Repko R.C., Scaife C.L., Adler D.G., Boucher K.M., Mulvihill S.J. (2014). Serum IGFBP2 and MSLN as diagnostic and prognostic biomarkers for pancreatic cancer. HPB Oxford.

[41] Ballehaninna U.K., Chamberlain R.S. (2012). The clinical utility of serum CA 19-9 in the diagnosis, prognosis and management of pancreatic adenocarcinoma: An evidence based appraisal. J. Gastrointest. Oncol..

[42] Mann D.V., Edwards R., Ho S., Lau W.Y., Glazer G. (2000). Elevated tumour marker CA19-9: Clinical interpretation and influence of obstructive jaundice. Eur. J. Surg. Oncol..

[43] Azizian A., Rühlmann F., Krause T., Bernhardt M., Jo P., König A., Kleiß M., Leha A., Ghadimi M., Gaedcke J. (2020). CA19-9 for detecting recurrence of pancreatic cancer. Sci. Rep..

[44] Fahrmann J.F., Schmidt C.M., Mao X., Irajizad E., Loftus M., Zhang J., Patel N., Vykoukal J., Dennison J.B., Long J.P. (2021). Lead-Time Trajectory of CA19-9 as an Anchor Marker for Pancreatic Cancer Early Detection. Gastroenterology.

[45] Kim B.J., Lee K.T., Moon T.G., Kang P., Lee J.K., Kim J.J., Rhee J.C. (2009). How do we interpret an elevated carbohydrate antigen 19-9 level in asymptomatic subjects?. Dig. Liver Dis..

[46] Tong Y., Song Z., Zhu W. (2013). Study of an elevated carbohydrate antigen 19-9 concentration in a large health check-up cohort in China. Clin. Chem. Lab. Med..

[47] Chang C.Y., Huang S.P., Chiu H.M., Lee Y.C., Chen M.F., Lin J.T. (2006). Low efficacy of serum levels of CA 19-9 in prediction of malignant diseases in asymptomatic population in Taiwan. Hepato-Gastroenterol..

[48] Kim J.-E., Lee K.T., Lee J.K., Paik S.W., Rhee J.C., Choi K.W. (2004). Clinical usefulness of carbohydrate antigen 19-9 as a screening test for pancreatic cancer in an asymptomatic population. J. Gastroenterol. Hepatol..

[49] Hong S., Song K.B., Hwang D.W., Lee J.H., Lee W., Jun E., Kwon J., Park Y., Park S.Y., Kim N. (2021). Preoperative serum carbohydrate antigen 19-9 levels predict early recurrence after the resection of early-stage pancreatic ductal adenocarcinoma. World J. Gastrointest. Surg..

[50] Goonetilleke K., Siriwardena A. (2007). Systematic review of carbohydrate antigen (CA 19-9) as a biochemical marker in the diagnosis of pancreatic cancer. Eur. J. Surg. Oncol..

[51] Ballehaninna U.K., Chamberlain R.S. (2011). Serum CA 19-9 as a Biomarker for Pancreatic Cancer—A Comprehensive Review. Indian J. Surg. Oncol..

[52] Winter K., Talar-Wojnarowska R., Dąbrowski A., Degowska M., Durlik M., Gąsiorowska A., Głuszek S., Jurkowska G., Kaczka A., Lampe P. (2019). Diagnostic and therapeutic recommendations in pancreatic ductal adenocarcinoma. Recommendations of the Working Group of the Polish Pancreatic Club. Prz. Gastroenterol..

[53] Steinberg W.M., Gelfand R., Anderson K.K., Glenn J., Kurtzman S.H., Sindelar W.F., Toskes P.P. (1986). Comparison of the sensitivity and specificity of the CA19-9 and carcinoembryonic antigen assays in detecting cancer of the pancreas. Gastroenterology.

[54] Liu C., Deng S., Jin K., Gong Y., Cheng H., Fan Z., Qian Y., Huang Q., Ni Q., Luo G. (2020). Lewis antigen-negative pancreatic cancer: An aggressive subgroup. Int. J. Oncol..

[55] Kim S., Park B.K., Seo J.H., Choi J., Choi J.W., Lee C.K., Chung J.B., Park Y., Kim D.W. (2020). Carbohydrate antigen 19-9 elevation without evidence of malignant or pancreatobiliary diseases. Sci. Rep..

[56] Ventrucci M., Pozzato P., Cipolla A., Uomo G. (2009). Persistent elevation of serum CA 19-9 with no evidence of malignant disease. Dig. Liver Dis..

[57] Steinberg W. (1990). The clinical utility of the CA 19-9 tumor-associated antigen. Am. J. Gastroenterol..

[58] Wolske K.M., Ponnatapura J., Kolokythas O., Burke L.M.B., Tappouni R., Lalwani N. (2019). Chronic Pancreatitis or Pancreatic Tumor? A Problem-solving Approach. RadioGraphics.

[59] Singh V.K., Yadav D., Garg P.K. (2019). Diagnosis and management of chronic pancreatitis: A review. JAMA.

[60] Braganza J.M., Lee S.H., McCloy R.F., McMahon M.J. (2011). Chronic pancreatitis. Lancet.

[61] Lowenfels A.B., Maisonneuve P., Cavallini G., Ammann R.W., Lankisch P.G., Andersen J.R., DiMagno E.P., Andren-Sandberg A., Domellof L. (1993). Pancreatitis and the Risk of Pancreatic Cancer. N. Engl. J. Med..

[62] Schima W., Böhm G., Rösch C.S., Klaus A., Függer R., Kopf H. (2020). Mass-forming pancreatitis versus pancreatic ductal adenocarcinoma: CT and MR imaging for differentiation. Cancer Imaging.

[63] Basso D., Plebani M., Fogar P., Panozzo M.P., Meggiato T., De Paoli M., Del Favero G. (1995). Insulin-like growth factor-I, interleukin-1 alpha and beta in pancreatic cancer: Role in tumor invasiveness and associated diabetes. Int. J. Clin. Lab. Res..

[64] Meggiato T., Plebani M., Basso D., Panozzo M., Del Favero G. (1999). Serum growth factors in patients with pancreatic cancer. Tumor Biol..

[65] Dahlem C., Barghash A., Puchas P., Haybaeck J., Kessler S.M. (2019). The insulin-like growth factor 2 mRNA binding protein IMP2/IGF2BP2 is overexpressed and correlates with poor survival in pancreatic cancer. Int. J. Mol. Sci..

[66] Gong Y., Zhang B., Liao Y., Tang Y., Mai C., Chen T., Tang H. (2017). Serum Insulin-Like Growth Factor Axis and the Risk of Pancreatic Cancer: Systematic Review and Meta-Analysis. Nutrients.

[67] Cousin S.P., Hügl S.R., Wrede C.E., Kajio H., Myers Jr M.G., Rhodes C.J. (2001). Free fatty acid-induced inhibition of glucose and insulin-like growth factor I-induced deoxyribonucleic acid synthesis in the pancreatic β-cell line INS-1. Endocrinology.

[68] Williams T., Berelowitz M., Joffe S.N., Thorner M.O., Rivier J., Vale W., Frohman L.A. (1984). Impaired Growth Hormone Responses to Growth Hormone–Releasing Factor in Obesity. A pituitary defect reversed with weight reduction. N. Engl. J. Med..

[69] Gao S., Sun Y., Zhang X., Hu L., Liu Y., Chua C.Y., Phillips L.M., Ren H., Fleming J.B., Wang H. (2016). IGFBP2 Activates the NF-κB Pathway to Drive Epithelial–Mesenchymal Transition and Invasive Character in Pancreatic Ductal Adenocarcinoma. Cancer Res..

[70] Dong J., Yu J., Li Z., Gao S., Wang H., Yang S., Wu L., Lan C., Zhao T., Gao C. (2021). Serum insulin-like growth factor binding protein 2 levels as biomarker for pancreatic ductal adenocarcinoma-associated malnutrition and muscle wasting. J. Cachex. Sarcopenia Muscle.

[71] Ge L., Pan B., Song F., Ma J., Zeraatkar D., Zhou J., Tian J. (2017). Comparing the diagnostic accuracy of five common tumour biomarkers and CA19-9 for pancreatic cancer: A protocol for a network meta-analysis of diagnostic test accuracy. BMJ Open.

[72] Van Manen L., Groen J.V., Putter H., Vahrmeijer A.L., Swijnenburg R.-J., Bonsing B.A., Mieog J.S.D. (2020). Elevated CEA and CA19-9 serum levels independently predict advanced pancreatic cancer at diagnosis. Biomarkers.

[73] Mehta S., Bhimani N., Gill A.J., Samra J.S., Sahni S., Mittal A. (2021). Serum biomarker panel for diagnosis and prognosis of pancreatic ductal adenocarcinomas. Front. Oncol..

[74] Hrabák P., Šoupal J., Kalousová M., Krechler T., Vočka M., Hanuš T., Petruželka L., Svačina S., Žák A., Zima T. (2022). Novel biochemical markers for non-invasive detection of pancreatic cancer. Neoplasma.

